# The Protective Effect of Docosahexaenoic Acid on Mitochondria in SH-SY5Y Model of Rotenone-Induced Toxicity

**DOI:** 10.3390/metabo15010029

**Published:** 2025-01-08

**Authors:** Britta Eggers, Jennifer Stepien, Anne-Katrin Reker, Svenja Esser, Kathy Pfeiffer, Magdalena Pawlas, Katalin Barkovits, Katrin Marcus

**Affiliations:** 1 Medizinisches Proteom-Center, Medical Faculty, Ruhr-University Bochum, 44801 Bochum, Germany; jennifer.stepien@rub.de (J.S.); anne-katrin.reker@rub.de (A.-K.R.); svenja.esser-p9c@rub.de (S.E.); kathy.pfeiffer@rub.de (K.P.); magdalena.pawlas@rub.de (M.P.); katalin.barkovits@rub.de (K.B.); 2 Medical Proteome Analysis, Center for Protein Diagnostics (PRODI), Ruhr-University Bochum, 44801 Bochum, Germany

**Keywords:** SH-SY5Y, docosahexaenoic acid, rotenone, mitochondria, proteomics

## Abstract

**Background:** Polyunsaturated fatty acids in particular omega-3 fatty acids, such as docosahexaenoic acid (DHA), are essential nutrients and components of the plasma membrane. They are involved in various processes, including synaptic development, functionality, integrity, and plasticity, and are therefore thought to have general neuroprotective properties. Considerable research evidence further supports the beneficial effects of omega-3 fatty acids, specifically on mitochondria, through their antioxidant and anti-apoptotic properties, making them an attractive addition in treatment options for neurodegenerative disorders in which mitochondrial alterations are commonly observed. However, precise information on the underlying protective mechanisms is still lacking. **Methods:** We utilized the most common neuronal cell line (SH-SY5Y) and induced mitochondrial oxidative stress through the addition of rotenone. To study the potential protective effect of DHA, the cells were additionally pre-treated with DHA prior to rotenone administration. By combining SILAC labeling, mitochondria enrichment, and subsequent proteomic analyses, we aimed to determine the capacity of DHA to alleviate mitochondrial oxidative stress in vitro and further shed light on the molecular mechanisms contributing to the proposed neuroprotective effect. **Results:** We confirmed a reduced cell viability and an increased abundance of reactive oxygen species upon rotenone treatment, DHA pre-treatment was shown to decrease said species. Additionally proteomic analysis revealed an increased expression of mitochondrial proteins in DHA pre-treated cells. **Conclusions:** With our study, we were able to define a potential compensatory mechanism by which the inhibition of complex I is overcome by an increased activity of the fatty acid beta oxidation in response to DHA.

## 1. Introduction

In the brain, polyunsaturated fatty acids (PUFAs), which are components of the plasma membrane, are involved in several essential processes, including enhanced synaptic development and functionality, and have effects on synaptic integrity and plasticity [1]. The main classes of PUFAs belong to omega-3 fatty acids, which include α-linolenic acid (ALA, 18:3 n-3), eicosapentaenoic acid (EPA, 20:5 n-3), docosahexaenoic acid (DHA, 22:6 n-3), and omega-6 fatty acids (n-6 PUFA) such as linoleic acid (LA, 18:2 n-6) and arachidonic acid (ARA, 20:4 n-6) [2]. Opposing characteristics of n-3 PUFA and n-6 PUFA have been described [3]. Most of the observed effects of n-3 PUFA are health-promoting, while n-6 PUFA has been shown to have disease-promoting and pro-inflammatory effects [3]. Therefore, therapeutic approaches tend to focus on the administration of the n-3 forms, which can be formed via the following pathways: (1) The essential precursor α-linolenic acid (ALA) is ingested with food, where ALA is then converted into unsaturated fatty acids, namely DHA or eicosapentaenoic acid (EPA), by synthesis with a series of desaturation, elongation, and β-oxidation reactions [4]; (2) Direct intake through seafood, especially fish or algae [2]. Several studies have already verified the immense potential of DHA. DHA represents the major PUFA in the brain, which accounts for over 90% of n-3 PUFA and 10–20% of total lipids in the brain [5]. DHA is mainly incorporated into phosphatidylethanolamine, phosphatidylserine and, to a lesser extent, phosphatidylcholine in synaptic terminals, mitochondria and endoplasmic reticulum [6]. Moreover, it can modulate cellular properties and physiological processes such as membrane fluidity, neurotransmitter release, gene expression, myelination, neuroinflammation, and neuronal growth [7]. Oguro et al. showed that DHA treatment in SH-SY5Y cells induces the activation of antioxidant genes (*CAT* and *SOD1*) [8]. Satyanarayanan et al. observed DHA-induced antioxidative and anti-inflammatory effects in SH-SY5Y cells through the activation of nuclear factor NF-κB response [9].

In addition, anti-inflammatory properties of DHA were observed by the induction of inhibiting inflammatory mediators, and DHA was found to have a general neuroprotective effect and to reduce DNA fragmentation thus adding to its anti-apoptotic properties [10,11]. Considerable research evidence further supports the beneficial effects of n-3 PUFA, specifically on mitochondria, with n-3 PUFA showing an impact on mitochondrial structure and function in an SH-SY5Y cell model [12]. In several studies, changes in mitochondrial dynamics have been observed through n-3 PUFA treatments [13], as well as an increase in mitochondrial biogenesis, i.e., the formation of new mitochondria [14,15]. The formation of new mitochondria through mitochondrial biogenesis is considered an interesting strategy to prevent or treat diseases associated with mitochondrial dysfunction, a typical hallmark of neurodegeneration [12].

Indeed, DHA treatment is thought to have a direct antioxidant and anti-apoptotic effect on mitochondria, but precise information on its influence on mitochondria-related redox aspects is lacking [12]. For example, Garrel et al. demonstrated that n-3 PUFA treatment (DHA and EPA) enhances the activity of antioxidative mitochondrial protein superoxide dismutase 2 (SOD2) in rat models [16]. Also, Luchtman et al. described an antioxidant effect of n-3 PUFA (EPA) mediated by its inhibition of the mitochondrial genes NADPH oxidase and cyclo-oxygenase-2 (COX-2) [11]. Administration of DHA in a rat model for Parkinson’s disease (PD) reduced MPTP-induced lesions, thereby reducing the extent of neuronal apoptosis [17].

A study analyzing the presence of long-chain PUFAs in PD patients detected a decreased abundance, particularly of DHA [18], stressing their importance to maintain the healthy physiological function of the brain. Therefore, supplementation with n-3 PUFA is already being used in patients to alleviate the symptoms of neurodegeneration. In the context of PD, n-3 PUFA intake has been shown to result in a decrease in apoptotic cells and an overall increase in dopaminergic neurons in substantia nigra pars compacta (SNpc) [19].

Overall, n-3 PUFA intake is very promising for protection against PD. However, the precise molecular mechanisms are still partially unknown. Since mitochondria play an essential role in regulating the key steps of cell death, it is important to study their involvement in DHA-induced protection. However, there is currently limited research on the alteration of mitochondrial proteins by DHA treatment on the molecular level, especially the level of proteins, since the majority of studies conducted so far have focused on changes in the lipid composition [20,21].

Thus, in this study, we aimed to elucidate the proposed and observed beneficial effects of DHA on mitochondrial proteins in one of the most widely used cell models for PD—the SH-SY5Y neuroblastoma cell line [22,23]. SH-SY5Y cells possess a catecholaminergic phenotype, meaning they can synthesize both dopamine (DA) and norepinephrine [23]. Differentiation is usually carried out by a combination of retinoic acid (RA) and *O*-tetradecanoyl-phorbol-13-acetate (TPA) treatment [23].

Furthermore, SH-SY5Y cells can be treated with specific drugs and/or genetic approaches to induce various PD-specific pathologies, resulting in a PD-like phenotype. The most commonly used drug that causes PD-like mitochondrial dysfunction is rotenone (Rot). The link between Rot and PD was identified in epidemiological studies on agricultural workers who used rotenone as a pesticide and displayed an increased risk of developing PD [24,25]. Several studies have already demonstrated that by the administration of Rot, a PD-like phenotype can be achieved in SH-SY5Y cells [26]. Rot is hydrophobic and enters the cell independently of transporters, where it inhibits complex I of the respiratory chain. This inhibition leads to impaired mitochondrial respiration and the increased production of reactive oxygen species (ROS), which result in oxidative stress in cells and mitochondria-mediated apoptosis [26]. Post-mortem studies on brain samples from PD patients have also shown that respiratory chain enzymes, particularly complex I, were reduced in both the relative number and amount of transcripts compared to the healthy subjects [27].

The analysis of Rot-treated SH-SY5Y cells has been performed in early studies and characterized stress-associated proteomic alterations. Zhou et al. described that Rot in SH-SY5Y cells modulates the expression of dynamin-related protein 1 (Drp1) and that its regulation of mitochondrial translocation mediates rotenone-induced cell death [28]. In the study by Zhang et al. a rotenone-induced change in the Akt/mTOR signalling pathway in SH-SY5Y cells was observed [29]. Dysregulation of the Akt/mTOR pathway is commonly reported in brains and dopaminergic (DAergic) neurons from PD patients and contributes to the loss of dopaminergic neurons in PD [29]. Aside from the alteration in the mitochondria, a previous study described a rotenone-induced modulation of proteins from the endoplasmic reticulum leading to ER stress [30,31].

In this study, we utilized this approach to determine the potential beneficial effects of DHA in the context of PD, by treating differentiated SH-SY5Y cells with Rot, which is known to cause the degeneration of dopaminergic neurons and alpha-synuclein inclusions, eventually leading to progressive parkinsonian motor deficits in humans and mammalian models, such as mice and rats [32]. With this in mind, we aimed to determine the capacity of DHA to alleviate PD-like symptoms, in particular neuronal degeneration in vitro, and further shed light on the molecular mechanisms contributing to the proposed neuroprotective effect.

## 2. Materials and Methods

### 2.1. Cell Culture and Treatment

Human neuroblastoma SH-SY5Y cells were obtained from DSMZ (German Collection of Microorganisms and Cell Cultures GmbH, Braunschweig, Germany) and cultured in DMEM/F-12 supplemented with 10% fetal bovine serum (FBS) and 1% penicillin/streptomycin (all Gibco, Thermo Fisher Scientific, Schwerte, Germany). During cell cultivation, the medium was changed every 2–3 days. Cells were differentiated into fully human neuron-like cells by adding 10 μM retinoic acid (RA) in the medium for 3 days followed by further incubation in a medium containing 80 nM 12-O-tetra-decanoylphorbol 13-acetate (TPA) for 3 days (all Merck, Sigma-Aldrich, Darmstadt, Germany). Cells were treated with 25 µM cis-4, 7, 10, 13, 16, 19-DHA for 48 h and/or 5.4 µM rotenone (Rot) (both Merck, Sigma-Aldrich, Saint Louis, MO, USA) for 24 h. As controls, differentiated SH-SH5Y were cultivated in TPA-supplemented media with 5.4 µM DMSO.

Cells subjected to mass spectrometric analyses, were cultivated in SILAC DMEM/F-12 media (Thermo Fisher Scientific) supplemented with 10% dialyzed FBS (Pan-Biotech, Germany), 100 U/mL penicillin/streptomycin (PAN-Biotech, Aidenbach, Germany), and either light (Lys0, Arg0, Merck, Sigma-Aldrich), medium (Lys4, Arg6, Thermo Fisher Scientific, Germany), or heavy labeled (Lys8, Arg10, Cambridge Isotope Laboratories, Inc., Cambridge, UK) amino acids (see Supplementary Figure S1). L-Glutamine was added as well as L-Proline to reduce arginine-to-proline conversion and incomplete SILAC labeling. The labeling efficiency was verified to be higher than >95% (see Supplementary Figure S1). For subcellular analysis, cells were labeled as follows: control (light label), Rot (medium label) and DHA + Rot (heavy label) (n = 5). Cell differentiation and treatment were conducted as described above.

### 2.2. Immunofluorescence Microscopy

For immunocytochemistry, cells were seeded in 24 well plates onto coverslips in a density of 30,000 cells/well. Prior to staining, cells were washed with PBS and fixed with 4% paraformaldehyde for 30 min. After 3 intensive washing steps with PBS, the cells were incubated with primary antibodies against βIII-tubulin (1:200, Abcam, Cambridge, UK) and vimentin (1:100, Santa Cruz Biotechnology, Dallas, TX, USA) overnight at 4 °C. Incubation with secondary antibodies Alexa Fluor 647 (1:1000, Invitrogen, Darmstadt, Germany) and Cy2 (1:200 Jackson Immunoresearch, Baltimore, PA, USA) was carried out at room temperature for one hour in the dark. Nuclei were counterstained with mounting medium containing DAPI (BIOZOL Diagnostica GmbH, Eching, Germany). The Olympus VS120 BX61VS virtual slide microscope and Olympus OlyVIA software 4.1 (build 2756) were used for fluorescence imaging. Stained cells were visualized at 20× or 40× magnification.

To determine the differentiation status of SH-SY5Y cells, five 500 × 500 μm sections per condition, distributed across the cell population, were acquired for semi-automatic counting and the subsequent manual review of cell subtypes present in the SH-SY5Y cell population that showed immunoreactivity using the βIII-tubulin (TUBB3) (marker for differentiated neurons) and vimentin (VIM) antibodies (marker for schwann cells). Cells displaying immunoreactivity for both markers were defined as intermediate cell type and were not considered for cell counting. Cell counting was performed in the image processing software Fiji (ImageJ, version 2.14.0/1.54f (National Institute of Health, Bethesda, MD, USA); Java 1.8.0_322 PlugIn OlympusViewer and NeuronJ), whereby the relative proportion of TUBB3- and VIM-positive cells was determined from the total number of cell bodies labeled with DAPI (approx. 650 cells). The cells were counted in an inverted 8-bit version to better delineate the cell structures represented in the respective channel. Descriptive statistics (mean values, standard deviation, and standard error) and visualization of the data were carried out in OriginPro (OriginLab Corporation, Northampton, MA, USA) utilizing one-sided ANOVA with subsequent Bonferroni correction, whereby an adjusted ANOVA *p*-value < 0.05 was considered statistically significant.

Successful differentiation was further determined by neurite length. In order to analyze neurite growth, the projections of 50 TUBB3-positive cells were measured from the previously scanned image sections using the Fiji Plugin NeuronJ (version 1.4.3) [33]. In preparation for neurite tracking in NeuronJ, imported images were processed and converted to an 8-bit version. Cell bodies with fully imaged neurites were marked and counted and corresponding projections were measured. Brightness and contrast were adjusted, and the background was subtracted to remove interfering artifacts prior to neurite tracking. The image was subsequently opened in NeuronJ, and projections were semi-automatically measured in the previously defined cells. A significant difference between the neurite lengths of undifferentiated and differentiated cells was determined via Student’s *t*-test, whereby a *p*-value < 0.05 was considered significant.

### 2.3. Dopamine Detection with High-Performance Liquid Chromatography

The SH-SY5Y cells (undifferentiated and differentiated, n = 3 each) were washed with PBS and gently scraped using a cell scraper. The cells were centrifuged at 400 g for 15 min, and the pellet was then resolved in 50 µL of 250 mM formic acid (FA). The extraction of metabolites was achieved by methanol (MeOH) precipitation. For this, 90 µL of MeOH/H_2_O (1:8 *v*/*v*) were aliquoted in reaction tubes and 10 µL of cell lysates were added. After mixing on the thermocycler at 4 °C for 5 min, the samples were centrifuged at 15,000× *g* at 4 °C for 5 min. The supernatant containing the metabolites was transferred into fresh tubes, and the pellet containing proteins were frozen again at −80 °C until further analysis. The supernatant was dried in the vacuum centrifuge at room temperature for 60 min. Finally, the samples were resuspended in 150 µL of FA-AsA buffer (1% formic acid, 0.5 mM ascorbic acid) for HPLC analysis. The dilation series of DA (Sigma Aldrich, Hamburg, Germany) was freshly prepared in FA-AsA buffer with concentrations of 0.005, 0.01, 0.05, 0.1, 0.5, 1, and 5 ng/µL DA.

All HPLC experiments were carried out on an Accquity UPLC system with an AccQ-Tag ULTRA™ reversed-phase C18 column with a diameter of 100 × 2.1 mm and a particle size of 1.7 µm (Waters, Milford, MA, USA). The UV detector was set to 280 nm. The injection volume was 2 µL for all samples and all measurements were performed in triplicates, while samples were kept at 10 °C in the autosampler. Elution was achieved with a gradient starting at 90% A and 10% B for the first 4 min, then increasing to 75% B and keeping this ratio for two minutes before linearly decreasing to 10% B again over 4 min. The total runtime was 12 min per sample. Elution buffer A was 0:1% FA in H_2_O and B was 0:1% FA in MeCN. The limit of detection (LOD) and the limit of quantification (LOQ) were calculated as the quotient of the standard deviation of the Y-intercept of three regression lines and the slope of the regression line. For the LOD, the signal had to be three times the quotient of standard deviation and slope for LOQ 10 times.

Calibration of the HPLC method was achieved utilizing the dilation series, as external standards of DA (concentration range: 5–0.005 ng/μL). Peaks were integrated, and the peak area plotted against concentration to generate an unweighted linear calibration curve. For the quantification of endogenous DA, the extracted metabolites of undifferentiated and differentiated SH-SY5Y cells were measured in triplicate and the peak area was integrated. The DA concentration was calculated with the equation of the regression line obtained from matrix matched calibration. In the final step, DA concentration was normalized on the protein concentration (in µg/µL) determined via Bradford assay, resulting in a defined DA concentration per µg protein (x ng DA/x µg protein).

### 2.4. Cell Viability Assay

For the sensitivity assessment of Rot and/or DHA treatment, a cell viability assay was performed according to the manufacturer’s instructions (CyQUANT XTT Cell Viability Assay kit, Thermo Fisher Scientific, Bremen, Germany). For that, SH-SY5Y cells were seeded at a density of 1 × 10^5^ in 96-well plates (Costar, Corning, NY, USA) with 100 µL cell suspension added to each well. After an adherence period of 24 h, cells were differentiated as described above. For single or combination treatments, Rot was added at concentrations of 0, 5, 10, 20, 50, and 100 µM for 24 h, 48 h, and 72 h and DHA at concentrations of 0, 10, 25, 50, and 100 µM for 24 h and 48 h; untreated control cells were added as controls.

For the blanks, three separate wells containing no cells, only cultivation medium, were prepared. For each treatment group, 6–8 replicates were chosen, corresponding to 6–8 wells. The absorbance of the unstressed control groups was considered as 100% cell viability. Data are presented as mean ± standard error of the mean. Data preparation was calculated with GraphPad Prism 9.0 (GraphPad Software Inc., San Diego, CA, USA). One-way analysis of variance (ANOVA) was used to identify significant differences between the experimental groups, and a subsequent Dunnett’s multiple comparisons test was used to compare the differences between each group; *p* < 0.05 was considered statistically significant.

### 2.5. Measurement of ROS Levels by DCFDA Assay

The intracellular level of ROS was determined using a cellular ROS detection assay kit (Abcam, Cambridge, UK), according to the manufacturer’s instructions, using a Multimode Plate Reader (Infinite M200 Pro, TECAN, Männedorf, Switzerland). SH-SY5Y cells were seeded in 96-well plates, with 10 × 10^4^ cells in 100 µL. After differentiation, cells were either not treated (Ctrl) or treated with 25 µM DHA for 48 h and/or 5.4 µM Rot for 24 h (n = 6 per each group, plus 3 blanks containing cell culture media only). The statistical analysis was carried out as described for the cell viability assay.

### 2.6. Western Blot Analysis

The Quantitative Smart Protein Layers (SPL) Western blotting system (NH DyeAGNOSTICS GmbH, Halle an der Saale, Germany) was used for the detection of quantitative differences between selected candidate proteins and used according to the manufacturer’s instructions, as described in [34,35]. In short, the total protein (20 µg) was pre-labeled with a red fluorescent fluorophore (700 nm detection wavelength), and a green fluorescence-labeled standard protein was spiked in size S =12.5 kDa or size L = 80 kDa (800 nm detection wavelength), enabling the error correction of differing sample loadings and data normalization between experiments. βIII-tubulin (1:5000, Abcam, Cambridge, UK), Cox UE IV (1:5000, Invitrogen, Carlsbad, CA, USA), Histone H3 (1:1000, Abcam, Cambridge, UK), were utilized as primary antibodies. Incubation was carried out overnight at 8 °C. Blots were washed three times with 1× TBS and incubated with a secondary antibody for one hour at room temperature (1:10,000, IRDye^®^ 800CW anti-Rabbit IgG Secondary, IRDye^®^ 800CW anti-mouse IgG Secondary). After washing, the Western blots were scanned at wavelengths of 700 and 800 nm using the Odyssey imaging system (LI-COR Biosciences, Lincoln, NE, USA). Quantification of the results was carried out using the manufacturer’s software (SPL LabImage Software, version 3.4.5), resulting in quantitative protein volumes (SPL normalized volume).

### 2.7. Mitochondria Isolation Using OptiPrep™ Gradient

SILAC-labeled SH-SY5Y cells were washed with PBS and gently scraped using a cell scraper. For each treatment, 10 cell culture dishes were pooled to enable a high mitochondrial yield and centrifuged at 400 g for 15 min. The following steps were performed at 4 °C. The pellet was resuspended in 750 µL RSB buffer (10 mM Tris/HCl, 10 mM NaCl, 14 mM CaCl_2_). After a 3 min incubation on ice, the cell suspension was centrifuged at 2400× *g* for 5 min. The resulting pellet was resuspended in RSB/MS buffer (420 mM Mannitol, 140 mM Sucrose, 10 mM Tris/HCl, 5 mM EDTA, protease inhibitors). For lysis, cells were passed through 24 G, 26 G, and 30 G needles attached to a 1 mL syringe. Cell lysates of the different experimental treatments were combined 1:1:1 depending on the protein concentration according to Bradford’s estimation. Afterward, 500 µL MS buffer was added to the combined cell lysates and centrifuged at 1500× *g* for 5 min. The supernatant was centrifuged at 10,000× *g* for 20 min and then resuspended in 750 μL SEM buffer (250 mM Saccharose, 10 mM MOPS-KOH, pH 7.2, 1 mM EDTA). To prepare a discontinuous density gradient, five gradient solutions were prepared by mixing gradient dilution buffer (250 mM Sucrose, 6 mM EDTA, 120 mM HEPES, pH 7.4) with OptiPrep™ (Sigma, Aldrich, Hamburg, Germany, 60% solution in H_2_O). The diluted OptiPrep™ density gradient solutions were carefully layered in a 7 mL ultracentrifuge tube (Beckman Coulter, Brea, CA, USA) in descending concentration, as follows: First, 30% OptiPrep™ solution, then 27%, 23%, 20%, 17%. Adding 250 µL OptiPrep™ solution to the samples resulted in a 15% concentration, which was added at the top of the density gradient. The tube was centrifuged at 30,000× *g* for 4 h at 4 °C using an ultracentrifuge Optima L80 XP (Beckman Coulter, CA, USA). After centrifugation, three distinct bands appeared in the gradient solution. These bands were collected and centrifuged at 16,000× *g* for 15 min. The mitochondria isolation via ultracentrifugation was carried out four times, resulting in 4 replicates per condition.

### 2.8. In-Gel Digestion of Proteins and Preparation of Peptides for LC-MS

In-gel digestion was carried out as described in [36], where 10 μg of protein per mitochondrial isolation (n = 4) were utilized. Resulting protein bands were excised out of the gel, destained, and subsequently dried via vacuum centrifugation. To start the digestion, trypsin was added (1:50 ratio of trypsin/protein) to the dry gel pieces. The digestion was carried out at 37 °C overnight and stopped by acidification. Peptides were eluted out of the gel using a combination of 0.05% trifluoroacetic acid/50% acetonitrile and a 15 min sonication step, which was repeated twice. Finally, the collected supernatants were dried in the vacuum centrifuge and resolved in 22 µL 0.1% TFA. Peptide concentration was determined via amino acid analysis [37]. For the analysis of the samples from mitochondrial enrichment, 400 ng were analyzed.

### 2.9. LC MS/MS Analysis

LC-MS/MS analysis was performed on Orbitrap Fusion Lumos Tribrid mass spectrometer (Thermo Fisher Scientific, Bremen, Germany) coupled to an Ultimate 3000 RSLC nano-LC system (Thermo Fisher Scientific), as described in [38]. In brief, the sample was loaded and concentrated on a trap column (Acclaim PepMap, 100, 100 μm × 2 cm, nanoViper, C18, 5 μm, 100 Å, Thermo Fisher Scientific, Bremen, Germany) within 7 min at a flow rate of 30 μL/min with 0.1% TFA. Peptide separation was performed on an analytical column (Acclaim PepMap RSLC, 75 μm × 50 cm, nanoViper, C18, 2 μm, 100 Å) at a flow rate of 400 nl/min with a 240 min gradient from 5 to 40% solvent B (solvent A: 0.1% FA, solvent B: 0.1% FA, 84% acetonitrile). Full MS scans were acquired from 350 to 1400 *m*/*z*, at a resolution of 120,000 at 200 *m*/*z*, for the detection of precursor ions (normalized 250%, 80 ms maximum injection time). Dynamic exclusion was set to 30 s and the cycle time to 2 s. MS/MS fragments were generated by high-energy collision-induced dissociation (HCD) with normalized collision energy (NCE) of 28. The fragments were analyzed in an Orbitrap analyzer with a 30,000 resolution at 200 *m*/*z* (normalized AGC 2000%, maximum injection time 80 ms). Resulting mass spectrometric raw data are available via ProteomeXchange with identifier PXD049302 [39,40].

### 2.10. Data Analysis

The generated *.raw files were processed with the MaxQuant software v.1.6.17.0, where the mitochondrial fractions (F1–3) were combined as one sample in the analysis output. Spectra were searched with the Andromeda search engine against the human UniProtKB/Swiss-Prot database (release 2021/11). Trypsin was chosen as the digestion enzyme, with a maximum of two missed cleavages allowed. The fixed modification was set to carbamidomethyl at cysteine residues. Methionine (M) oxidation was included as a variable modification. False discovery rate (FDR) thresholds were set to 0.01 on peptide spectrum matching and protein level. Identification of co-fragmented peptides (second peptides) and match between runs (MBRs) features were enabled. The minimum score for modified peptides was set to 40. Arg6/Lys4 were set as the medium and Arg10/Lys8 as heavy labels, and the re-quantify option was enabled. The statistical analysis was carried out using Perseus (version 1.6.10.43). Raw intensities of all three channels were normalized using median subtraction and were further utilized for the determination of significantly regulated proteins. For that, the normalized intensities were log2 transformed, and a minimum of 70% of valid values was used to filter identified proteins. To identify the significantly differential proteins between the sample groups, Student’s *t*-test was applied (two-tailed, unpaired, adjustment according to Benjamini–Hochberg (q-value)). Ratios (fold changes) between the experimental groups were calculated using mean values, and proteins displaying a q-value < 0.05 were considered to be significantly differentially expressed. Pathway and gene annotation enrichment analysis was carried out using DAVID Bioinformatics Resources 6.8 [27,28]. For that, the functional annotation tool was chosen. Uniprot accession of proteins were uploaded. Here, only the first accession for the protein group results was taken to enable accurate analysis. The Uniprot ID identifier was set, and the list type was set to gene list. As a background, the whole *Homo Sapiens* genome was chosen. Cellular Compartment was chosen for GO term enrichment studies. The fold enrichment scores as well as *p*-value were utilized to determine a significant enrichment.

## 3. Results

### 3.1. Differentiated Dopamine Producing SH-SY5Y Cells as a Neuronal Cell Model

SH-SY5Y cells represent one of the most common and basic models to study neurons. However, to obtain meaningful results, the following two preconditions must be met: first, the cells used must be differentiated in order to exhibit properties as similar as possible to neurons; second, the cells should produce a specific neurotransmitter depending on the neuronal subtype studied.

**Figure 1:**
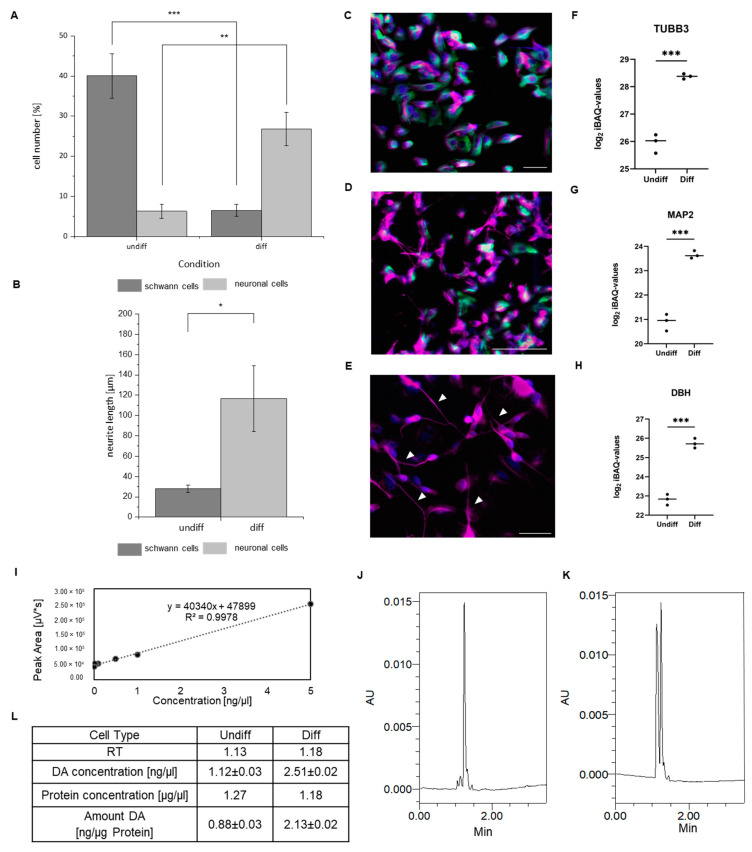
Morphological and metabolic changes of differentiated SH-SY5Y cells. (**A**) Differentiated (diff) SH-SY5Y cells display an increased number of neuronal (N) cells (adjusted one-way analysis of variance (ANOVA) *p*-value ** < 0.01) and a decreased number of Schwann cell-like (S) cells (adjusted ANOVA *p*-value *** < 0.001) than undifferentiated (undiff) cells. Five 500 × 500 μm sections of (**C**) undiff and (**D**) diff SH-SY5Y (20× magnification, scale 50 µm) distributed across the cell population, were acquired for manual counting of cell subtypes present in the SH-SY5Y cell population that showed immunoreactivity using tubulin β-3 chain (TUBB3, in pink, marker for diff neurons) and vimentin (VIM, in green marker for Schwann cells) antibodies. The relative proportion of TUBB3- and VIM-positive cells was determined from the total number of cell bodies labeled with DAPI (approx. 650 cells). (**B**) Diff cells exhibit a significant increase in neurite length (*p*-value * < 0.05) compared to undiff cells. (**E**) SH-SY5Y cells (40× magnification, scale 50 µm) show significantly elongated and branched neurites visualized by TUBB3 (in pink, marked with arrows, Student’s *t*-test *p*-value < 0.05). (**F**–**H**) Quantification of protein markers during differentiation of SH-SY5Y cells (n = 3). Abundance of the neuronal marker proteins (**F**) tubulin β-3 chain (TUBB3), (**G**) and microtubule-associated protein-2 (MAP2), as well as H. dopamine beta-hydroxylase (DBH) is measured by LC-MS/MS. Plots display log2 iBAQ intensities of all replicates and the mean value is marked. After differentiation, the level of all neuronal markers increased significantly. Statistical analysis was performed by *t*-test and Benjamini-Hochberg correction. *p*-value *** < 0.001. (**I**) Calibration curve of synthetic dopamine (DA). Concentration of synthetic DA with 0.005, 0.01, 0.05, 0.1, 0.5, 1, and 5 ng/µL measured in triplicates using HPLC-based detection. R^2^ is 0.9978. Based on the chromatograms ((**J**) DA in undifferentiated (n = 3) and (**K**) in differentiated SH-SY5Y cells (n = 3)). (**L**) DA concentration of undiff and diff SH-SY5Y cells was determined with the equation of the regression line of the DA calibration curve displayed in (**F**). Measured concentrations, retention time (RT) for DA on HPLC, and protein concentrations (Bradford) as means of three replicates are given in the table. The DA quantity was further normalised in relation to the protein concentration and revealed a substantial increase in DA in differentiated cells.

In our study, we aimed to elucidate the molecular, potential neuroprotective effect of DHA in a PD cellular model. We have created a neuronal cellular model resembling dopaminergic neurons based on the neuroblastoma cell line SH-SY5Y. Dopaminergic neurons are highly susceptible to ROS stress, and further Rot exposure is known to be a major factor in developing PD-like symptoms. To verify the cell differentiation of SH-SY5Y cells induced by retinoic acid (RA) and 12-O-tetradecanoyl-phorbol-13-acetate (TPA) treatment to a mature neuron-like phenotype, cells were initially investigated morphologically. Since SH-SY5Y cells are composed of two distinct cell types neuronal-like (N)-type cells and Schwann cell-like (S) type cells, differentiation was verified by counting the number of N- and S-type cells in undifferentiated (undiff) and differentiated (diff) cells (see Figure 1A,B,D). Additionally the lengths of neurites was assessed before and after differentiation (see Figure 1B,E). In parallel, the differentiation marker proteins were checked by MS-based label-free quantitative proteome analysis, and the DA production of the cells was verified using µHPLC analysis.

Overall, the dopaminergic phenotype of the cells could be confirmed. As expected, the differentiated SH-SY5Y cells showed morphological changes after RA and TPA treatment, displaying a 21.5% increase in N-type cells (adjusted one-way ANOVA *p*-value < 0.01) and a 33.5% decrease in S-type cells (adjusted one-way ANOVA *p*-value < 0.001, see Figure 1A,C,D), as well as significantly elongated and branched neurites (Student’s *t*-test *p*-value < 0.05, see Figure 1B) visualized by tubulin β-3 chain (TUBB3) staining, a marker for mature neurons (see Figure 1E indicated with white arrows).

On the proteome level, several marker proteins for a neuronal phenotype were significantly increased in differentiated SH-SY5Y cells, confirming a successful differentiation (see Figure 1F,G). Among them were the two major differentiation markers TUBB3 [41] and microtubule-associated protein-2 (MAP2) [42]. Further, an increase in dopamine beta-hydroxylase (DBH) was observed, which is considered as a marker for catecholaminergic phenotypes (based on Kovalevich et al.) (see Figure 1H). For the evaluation of DA production, a concentration series of synthetic DA (0.005–5 ng) was measured for quantification (see Figure 1I), and differentiated cells produced a substantially higher amount of DA, respectively, 2.13 ng/µg (see Figure 1J–L), compared to their undifferentiated counterpart (0.88 ng/µL DA).

### 3.2. Rotenone Decreases and DHA Potentially Increases the Cell Viability of Differentiated SH-SY5Y Cells

A third essential prerequisite to study the influence of substances in a cellular model is the determination of optimal treatment conditions, namely duration of treatment as well as substance concentration. To investigate the individual effect of Rot or DHA as well as the combination of both substances on the differentiated SH-SY5Y cells, the following four independent groups were defined: (1) control cells (Ctrl, without any treatment); (2) Rot treatment; (3) DHA treatment; (4) pre-treatment with DHA before Rot treatment.

**Figure 2:**
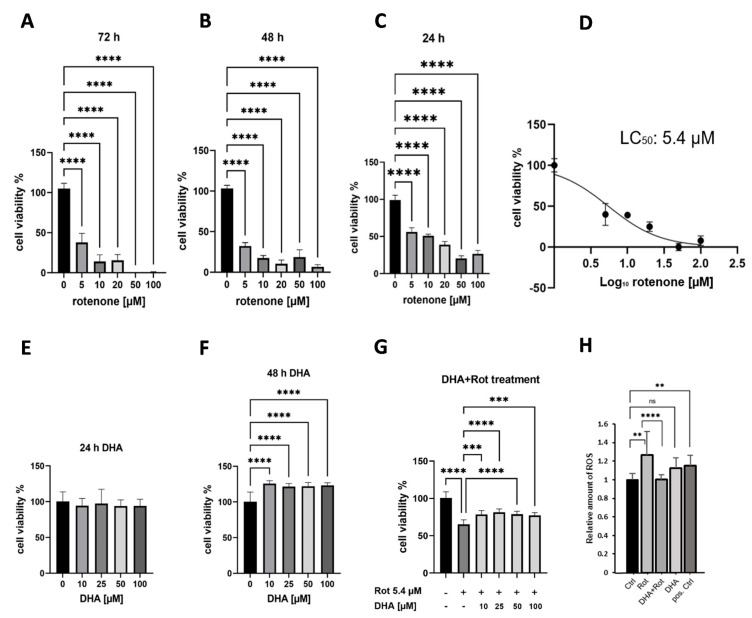
Effect of Rot and DHA treatment on cell viability and ROS production. To identify significant differences between experimental groups, one-way analysis of variance (ANOVA) and a subsequent Dunnett’s multiple comparisons test was used to compare the differences between each group. For all comparisons, *p* < 0.05 was considered statistically significant. SH-SY5Y cells were treated with Rot (for (**A**) 72 h (**B**) 48 h (**C**) 24 h) or DHA (for (**E**) 24 h (**F**) 48 h) in concentrations from 5 to 100 µM (**** *p* < 0.0001 vs. Ctrl) (for all treatment groups n = 6). The results are displayed as the percentage of cell viability of the untreated control group (Ctrl) in a bar chart representing the mean ± SD (n = 6). The Rot treatment resulted in a dose-depending decrease in cell viability compared to Ctrl in all tested concentrations already after 24 h (**** *p* < 0.0001). (**D**) Dose–response curve of Rot concentrations from 0 to 100 µM during treatment time of 24 h (n = 8). The calculated LC_50_ value is 5.4 µM for Rot (**E**) DHA treatment showed no significant effect after 24 h. (**F**) An increase in cell viability by 25% was observed already at a concentration of 10 µM after 48 h. (**G**) Pre-treatment with DHA led to a reduced Rot-induced toxicity (5.4 µM Rot for 24 h) for all tested DHA concentrations (10 µM, 25 µM, 50 µM, and 100 µM DHA increased cell viability to 73% (*p* < 0.001), 80% (*p* < 0.001), 75% (*p* < 0.01) and 71% (*p* < 0.001), respectively). Bar chart represents mean ± SD, vs. Ctrl/Rot *** *p* < 0.001, **** *p* < 0.0001 vs. Rot (n = 6). (**H**) ROS production. SH-SY5Y cells were treated with 5.4 µM Rot for 24 h (Rot), 25 µM DHA for 48 h (DHA), 25 µM DHA for 48 h, or 5.4 µM Rot for 24 h (DHA + Rot), as well as 55 mM TBHP for 4 h as a positive control (pos. Ctrl). ROS production was determined by DCFDA / H2DCFDA assay. The determined average Ctrl value was set to 1. Bar chart represents mean ± SD, **** *p* < 0.001 and ** *p* < 0.01, vs. Ctrl (n = 6) or not significant (ns). Rot-treated cells showed a significant increase in ROS content, while a pre-treatment with DHA reduced the ROS level. DHA treatment alone caused no change in ROS content.

To study the potential of DHA to alleviate Rot-induced cellular stress, it is essential to verify the detrimental effect of Rot treatment first. Thus, the appropriate concentrations of Rot and treatment duration were determined with the goal to define the mean lethal concentration (LC50), which was verified by cell viability assays. For this, differentiated SH-SY5Y cells were treated with different concentrations of Rot, ranging from 0 to 100 µM for 24 h, 48 h, and 72 h (see Figure 2A–C). Additionally, non-treated cells were seeded and cultivated as Ctrl.

A dose-dependent decrease in cell viability was observed after 24 h of treatment, while the high concentrations of 20, 50, and 100 µM led to a strong reduction in cell viability below the LC50 (see Figure 2C). After 48 h and 72 h Rot treatment, cell viability decreased below 50% at all investigated concentrations, which was below the target LC50 value (*p* < 0.001, Bonferroni after two-way ANOVA) (see Figure 2A,B). The concentrations of 5 and 10 µM were within the target range with 56% and 48% cell viability, respectively. To calculate the exact LC50 value, the 24 h treatment time was selected to perform a dose–response curve analysis (see Figure 2D). The concentration-dependent toxicity of Rot was confirmed with an LC50 value at 5.4 µM. This concentration was further used in subsequent experiments to induce Rot-dependent cellular stress.

Next, we aimed to confirm that DHA has a positive effect on the neuronal cell culture. Cells were treated with DHA alone for 24, 48, and 72 h at different concentrations (10 μM to 100 μM) and cellular viability was determined in comparison to untreated cells. (see Figure 2E). Treatment with DHA for 24 h showed no significant effect independent from the respective concentration (see Figure 2E). After 48 h of treatment, an increase in cell viability for all concentrations was observed (*p* < 0.0001, Bonferroni two-way ANOVA, see Figure 2F). These results support the proposed positive effect of DHA on differentiated SH-SY5Y cells already at a DHA concentration of 10 µM (potential increase in cell viability by 25%). A similar potential increase in cell viability was observed for the remaining evaluated DHA concentrations.

To finally check a possible balancing effect of DHA on Rot-stressed cells, a combined treatment of DHA and Rot was applied. As expected from the former experiments, a Rot concentration of 5.4 µM decreased cellular viability to 65% (see Figure 2G). However, according to our hypothesis, pre-treatment with DHA led to a reduced Rot-induced toxicity for all tested DHA concentrations. Pre-treatment with 10 µM, 50 µM, and 100 µM DHA increased cell viability to over 70%, respectively. Highest cell viability was achieved by the addition of 25 µM DHA, reaching an increase in viability up to 80% (*p* < 0.001).

Our findings are summarized as follows:

- The known toxic effect of Rot on differentiated SH-SY5Y cells has been confirmed;
- treatment of cells with DHA alone potentially leads to an increase in cell viability;
- DHA compensates for Rot-induced toxicity when treated in combination.

Optimal concentrations and treatment duration (i.e., DHA with 25 µM for 48 h and Rot with 5.4 µM for 24 h) were further utilized for the subsequent elucidation of molecular mechanisms to explain the potential of DHA for the rescue of the toxic effect of Rot.

### 3.3. Determination of ROS Production During DHA and/or Rot Treatment via ROS Detection Assay

DHA is known to have antioxidant properties [8,9]. Thus, we hypothesized that DHA may alleviate the Rot-induced accumulation of reactive oxygen species (ROS) in mitochondria, preventing mitochondrial alterations. To verify these assumptions in vitro, we performed an ROS detection assay. Cells were treated with the pre-determined optimal conditions for DHA and Rot. As a positive control, cells were treated with 50 µM tert-butyl hydrogen peroxide (tbHP), which acts as an exogenous inducer of oxidative stress.

As expected, the positive control tbHP induced an increase in cellular ROS (*p* < 0.01). Treatment with Rot alone should also increase the ROS levels in SH-SY5Y cells, which was proven for the selected Rot concentration (*p* < 0.01). The DHA pre-treatment decreased the Rot-induced accumulation of ROS, nearly to the control level (*p*  <  0.001), indicating a reduction in oxidative stress. Cells exclusively treated with DHA showed no difference to the Ctrl SH-SY5Y cells (summarized in Figure 2H).

### 3.4. Molecular Effects of Docosahexaenoic Acid and/or Rotenone Treatment on Isolated Mitochondria of Differentiated SH-SY5Y Cells

We wanted to determine the molecular changes that occur as a result of treatment with Rot alone compared to DHA pre-treated cells to potentially identify the specific mechanisms that contribute to the above-mentioned beneficial effects of DHA pre-treatment on cell viability and ROS production. Since Rot is known to inhibit complex I activity, we optimized the detection of mitochondrial proteins by enrichment via subcellular fractionation. However, subcellular fractionation requires a high number of cells to obtain visible fractionation in the gradient, which results in high effort and cost when applying this method to experimental groups and replicates. Therefore, we combined a multiplexing stable isotope labeling by amino acids in cell culture (SILAC) approach with subcellular fractionation. This allowed for a multiplexing of the different experimental groups (Ctrl, Rot, and DHA + Rot), thereby decreasing the number of cells needed per treatment. For this purpose, SH-SY5Y cells treated with DHA and/or Rot were cultured in three different conditioned media (see Supplementary Figure S1A). With this approach, cells can be combined (1:1:1), processed together, and measured in one run by mass spectrometry. To determine the labeling efficiency (minimum 95% labeled amino acids), a label efficiency study was conducted in parallel to the DHA- and/or Rot-treated cells, where all the samples showed a required labeling efficiency of above 97% and were suitable for further analysis (see Supplementary Figure S1B,C).

Subcellular fractionation resulted in three potential mitochondrial-enriched fractions. As a prerequisite, the enrichment of mitochondrial proteins was verified utilizing intensity-based absolute quantification (iBAQ) values. Indeed, subcellular fractionation doubled the number of quantified mitochondria-associated proteins compared to unfractionated whole cells in two of the three analyzed fractions. The depletion of other organelle markers was additionally verified via Western blot analysis (Supplementary Figure S1D–J).

### 3.5. Subcellular Proteomic Analysis to Investigate the Effect of DHA on Rot-Induced Toxicity in SH-SY5Y Cells

**Figure 3:**
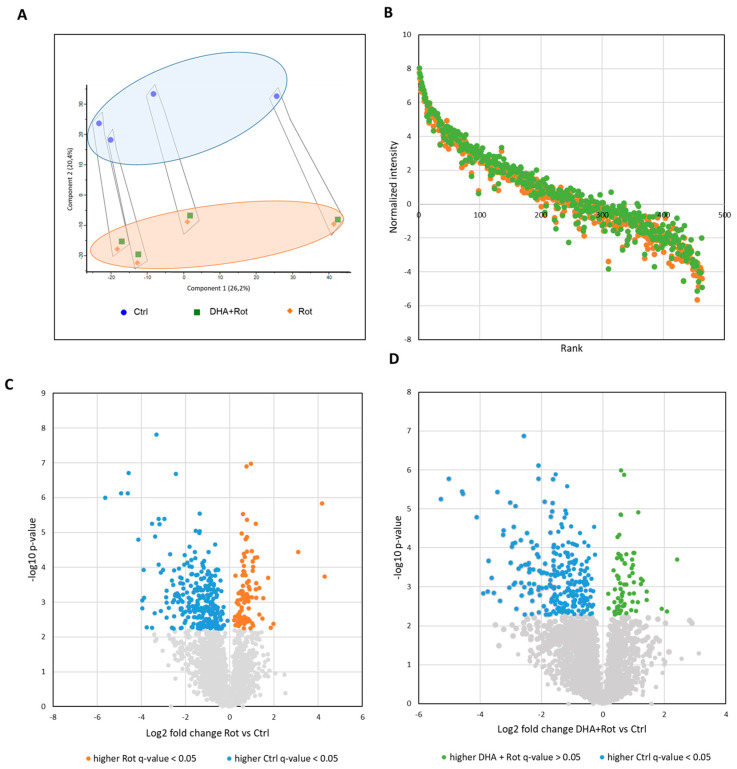
Quantitative assessment of proteomics changes. (**A**) Principal component analysis of enriched mitochondrial fractions (n = 4) of control cells (Ctrl, blue circle), DHA pre-treated + rotenone-treated cells (DHA + Rot, green square), and rotenone-treated cells (Rot, orange diamond). Grouping of samples can be observed for Component 1 (highlighted in a black border) indicating technically derived differences between the different density gradient enrichment replicates. On Component 2, the grouping of samples into experimental groups is highlighted. Ctrl cells form a distinct cluster (light blue circle) apart from DHA + Rot and Rot cells (light orange circle). (**B**) Ranking plots of mitochondrial protein abundances from Rot- and DHA + Rot-treated SH-SY5Y cells. Plots show ranked normalized intensities of Rot/Ctrl and DHA + Rot/Ctrl of all identified mitochondrial proteins. Proteins from the Rot treatment are displayed in orange, and DHA + Rot treatment in green points. The comparison revealed that most mitochondrial proteins are higher in DHA + Rot-treated cells than in solely Rot-treated cells. (**C**) Volcano Plot displaying differentially regulated proteins (Student’s *t*-test, two-tailed, unpaired, adjustment according to Benjamini–Hochberg (q-value), differentially regulated proteins are defined by q-value < 0.05) between Rot (orange) and Ctrl (blue). (**D**) Volcano Plot displaying differentially regulated proteins (Student’s *t*-test, two-tailed, unpaired, adjustment according to Benjamini–Hochberg (q-value), differentially regulated proteins are defined by q-value < 0.05) between DHA + Rot (green) and Ctrl (blue).

To investigate the underlying potential of DHA to alleviate Rot-induced toxicity specifically in mitochondria, quantitative protein analysis of enriched mitochondrial fractions was performed. In this analysis, a total of 4044 proteins were identified, of which 3017 proteins were used to determine significantly regulated proteins (see Supplementary Table S1). To gain an initial overview on global proteomic differences within our experimental groups, we performed a principal component analysis (see Figure 3A). Initially, we found that the density gradient had an influence on the distribution of the samples. However, this can be neglected as all experimental groups were included in each enrichment experiment due to our innovative SILAC approach, eliminating technical-based influences. As expected, Rot-treated cells formed a distinct cluster opposite from Ctrl cells, indicating a profound difference in their global proteome. Additionally, samples pre-treated with DHA prior to Rot administration formed a cluster in close proximity to the Rot group, potentially indicating only a mild protective effect. Since Rot is a potent inhibitor of complex I, we aimed to analyze mitochondrial proteins specifically. Thus, all quantified proteins were compared with the MitoCarta 3.0, identifying 463 closely associated proteins (see Supplementary Table S2). To gain the first overview of a potential therapeutic effect of DHA specifically on mitochondria, a ranking plot of all quantified mitochondrial proteins was created (see Figure 3B), displaying intensity levels of all identified mitochondrial proteins in either Rot- or DHA + Rot-treated cells. Interestingly, 80% of identified mitochondrial proteins had a higher intensity in DHA + Rot, compared to Rot-treated cells. Thus, we can speculate that DHA pre-treatment may have a global effect on mitochondrial proteins. Next, we aimed to study the effect of both treatments compared to untreated cells. To do so, we performed a quantitative comparison, revealing 366 proteins being differentially expressed between Ctrl and Rot-treated cells (Rot/Ctrl, see Figure 3C and Supplementary Table S3), as well as 322 proteins in DHA + Rot vs. Ctrl cells (DHA + Rot/Ctrl, see Figure 3D).

**Figure 4:**
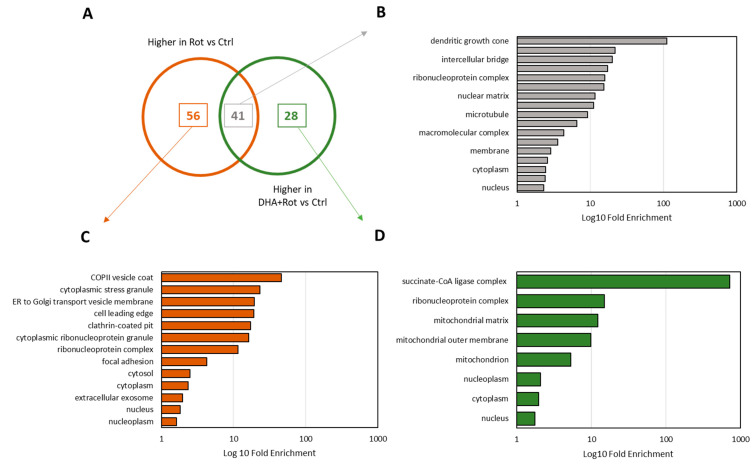
Functional assessment of differential proteins. (**A**) Overlap analysis of proteins being of higher abundance in Rot-stressed cells (n = 4, in red) and DHA + Rot-treated cells (n = 4, in green) compared to the Ctrl (n = 4) (Student’s *t*-test, two-tailed, unpaired, adjustment according to Benjamini–Hochberg (q-value), differentially regulated proteins are defined by q-value < 0.05). Common proteins are displayed in grey. (**B**) GO Term enrichment analysis based on cellular compartments of common upregulated proteins in both treatment groups compared to Ctrl. (**C**) GO Term enrichment analysis based on cellular compartments of uniquely upregulated proteins in Rot-treated cells compared to Ctrl. (**D**) GO Term enrichment analysis based on cellular compartments of uniquely upregulated proteins in DHA + Rot-treated cells compared to Ctrl.

Although the number of differential proteins were found to be similar between both comparisons, we wondered whether both treatments induced similar changes within the proteome or if DHA pre-treatment enabled the upregulation of potential protective mechanisms. Hence, we cross-compared the proteins of higher abundance in Rot-treated cells (Rot vs. Ctrl) and the proteins being increased in DHA + Rot cells (DHA + Rot vs. Ctrl). Of 97 proteins being of higher abundance in Rot cells (vs. Ctrl) and 69 proteins being of higher abundance in DHA + Rot cells (vs Ctrl), an overlap of 41 proteins could be detected (see Figure 4A and Supplementary Table S3). To gain a deeper understanding of Rot- or DHA + Rot-specific influences, we subsequently conducted a gene ontology enrichment analysis based on the cellular compartments (CC) of selected proteins (see Figure 4B–D and Supplementary Table S4). Common proteins being upregulated in comparison to the Ctrl in both treatments were found to be associated with the dendritic growth cone, ribonucleoprotein, and macromolecular complexes, the nuclear matrix, microtubules, the membrane, cytoplasm, and the nucleus. Proteins being solely upregulated in response to Rot treatment, however, were mainly affiliated with different types of vesicles and granules, such as COPII vesicles, exosomes, and stress granules. As already hypothesized, proteins being uniquely of higher abundance in DHA pre-treated cells displayed a close connection to mitochondria. Of 28 uniquely regulated proteins, 11 proteins were associated with terms like mitochondrial matrix, mitochondrial outer membrane and mitochondrion, indicating the potential of DHA to alleviate Rot-induced toxicity on mitochondria in particular.

To investigate this potential protective mechanism, we annotated all mitochondrial-associated proteins (based on GO Term analysis) being of higher abundance in DHA + Rot cells with regard to their mitochondrial localization and/or function (see Table 1). It was notable that the majority of proteins were involved in metabolic processes, providing ATP under oxidative conditions, particularly the citric acid cycle and the fatty acid beta oxidation.

**Figure 5:**
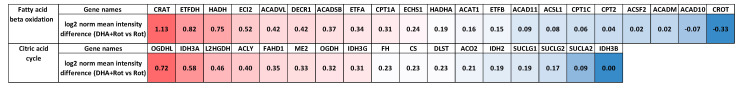
Intensities of pathway-associated proteins. Heat map displaying the difference of log 2 normalized mean protein intensities of DHA + Rot- vs. Rot-treated cells (n = 4 each) for proteins of the fatty acid beta oxidation and the citric acid cycle, based on MitoCarta 3.0. Differences in mean intensities are highlighted utilizing a color gradient and were sorted, respectively, (high differences in mean values in red, small differences in mean values highlighted in blue).

To substantiate our findings and potentially reveal a protective mechanism of DHA, we determined the abundance levels of all identified proteins associated with the fatty acid beta oxidation and the citric acid cycle for both treatments. For that, we calculated the mean log2 intensity of associated proteins for Rot-treated and DHA + Rot-treated cells and subsequently subtracted the resulting values (see Figure 5). It was obvious that the majority of proteins involved in both pathways displayed higher intensities in DHA + Rot-treated cells (see Figure 5, indicated as positive values > 0), among them were several proteins which were found to be exclusively regulated in DHA + Rot cells vs. Ctrl cells, such as ECHS1, a key protein of the fatty acid beta oxidation pathway.

Based on all previous results, which indicate that DHA has a positive effect on the abundance of mitochondrial proteins, we verified an increased abundance of the mitochondrial marker protein cytochrome c oxidase (COX IV) in DHA-treated cells via quantitative Western blot analysis (see Supplementary Figure S2). Confirmatively, SH-SY5Y cells treated with Rot displayed the lowest abundance of COXIV. In contrast, cells pre-treated with DHA followed by Rot treatment showed an increase in COX IV, adding to the hypothesis that DHA supplementation may offer mitochondria-modulating properties.

## 4. Discussion

With this study, we aimed to gain insights into the proteomic changes in differentiated SH-SY5Y cells in response to DHA and Rot treatment. Differentiated SH-SY5Y cells were treated with DHA and/or Rot, and the effects were either investigated using cell culture assays, morphological characterization, or global proteome analysis of mitochondria-enriched fractions. The performed studies led to the following main results:

1. Treatment with Rot led to reduced cell viability and ROS production accompanied by an increased abundance of stress granule proteins, indicating acute cell stress;
2. DHA treatment potentially increased the cell viability of differentiated SH-SY5Y cells and did not influence ROS levels;
3. DHA pre-treatment was shown to have a beneficial effect by improving cell viability in Rot-stressed cells and reducing Rot-induced ROS accumulation, supported by an increase in the abundance of proteins associated with several mitochondrial processes.

Synaptic development, function, integrity, and plasticity in the brain are depending on the supply of unsaturated fatty acids, as they are essential components of the plasma membrane [1,6]. Supplementation of omega-3 fatty acids, such as DHA, is thought to have health-promoting effects and is therefore the focus of therapeutic approaches. However, the exact mechanisms of how DHA is beneficial for neuronal survival are not fully elucidated yet.

In cell culture experiments, the different effects of DHA have been described as influencing physiological processes like membrane fluidity [7,18,43] or the exhibition of antioxidant activity by modulating the gene expression of several genes important for cellular antioxidative defense [8,9,10,12,19,44]. This makes DHA an interesting therapeutic agent, potentially alleviating symptoms present in neurodegenerative conditions, such as an increased production of ROS, leading to neuroinflammation. Indeed, a protective effect of DHA in the context of PD has been described in many studies; however, the precise role of DHA in the context of ROS accumulation in mitochondria and oxidative stress has not been fully elucidated. Thus, in this study, we utilized a rotenone-based in vitro neuronal model of PD (SH-SY5Y cells) to study the potential neuroprotective effect of DHA on the molecular level. SH-SY5Y cells are widely and commonly used to conduct functional studies focused on neurons. In PD research, SH-SY5Y cells are utilized, since they contain essential characteristics of catecholaminergic neurons and thus can be differentiated into a dopaminergic phenotype [23,45]. Further, by introducing common environmental toxins, such as rotenone or MPP^+^, a PD-like phenotype can be achieved, leading to increased ROS formation and eventually neuronal death [46]. However, we need to stress that there are several limitations utilizing SH-SY5Y cells, as follows: First, from a molecular point of view, targeted differentiation into one specific fully differentiated neuronal phenotype is limited and cannot be achieved to a full extent. Further SH-SY5Y cells derive from a neuroblastoma. Thus, presented results should be verified in a system closer resembling the in vivo environment, e.g., differentiated LUHMES cells and iPSC cell lines differentiated into dopaminergic neurons [47]. Nevertheless, we are convinced that the findings presented here are an important basis for further analyses in more appropriate cell models, since the enrichment of organelles requires a high number of cells, which is often not feasible in iPSC-based cell culture systems.

Confirmation of the functionality of the established model was evaluated in several steps, whereby optimal treatment conditions for Rot, DHA, and DHA + Rot were determined via cell viability and ROS assay. Further, proteomic changes induced by either Rot or DHA only were analyzed to determine general proteomic changes in response to treatment.

As expected and shown in several studies [48,49], Rot-treated cells displayed a decreased cell viability and ROS detection assay revealed a significant increase in ROS production.

DHA-treated cells instead showed an increase in cell viability after 48 h of treatment. This DHA-induced increase in cell viability may imply enhanced proliferation; however, since the neuronal cells utilized were differentiated, this observation could to point towards a change in mitochondrial enzyme activities, as the cell viability assay used was based on the metabolic reduction of XTT, a tetrazolium derivative, into a water-soluble orange product by the enzyme mitochondrial dehydrogenase. Indeed, a DHA-induced increase in the abundance of mitochondrial proteins was indicated by Western blot analysis. Nevertheless, with our proteomics-based approach, we cannot make any assumptions on the enzymatic activities, which may provide a more insightful view, on the potential of DHA modulating mitochondrial protein abundance and/or activity.

Additionally, pre-treatment with DHA prior to Rot administration resulted in a significant increase in cell viability compared to Rot treatment alone, suggesting a protective effect of DHA. Determination of ROS production supported a protective effect of DHA even further, as DHA pre-treatment significantly reduced Rot-induced ROS production in SH-SY5Y cells. As previously mentioned, the antioxidant strength of DHA has already been proven in a large number of studies [50,51,52]. Among them, a dietary study, identifying a profound effect of PUFAs with respect to mitochondrial membrane characteristics and functions [53]. Further, a direct correlation between DHA supplementation and the expression of antioxidant enzymes such as superoxide dismutases, catalases [54], and glutathione peroxidases [55] was established. The authors noted that DHA was particularly efficient in triggering the upregulation of Gpx4 gene, which encodes for the nuclear, cytosolic, and mitochondrial isoforms of phospholipid-hydroperoxide glutathione peroxidase (PH-GPx/GPx4), the main enzyme protecting cell membranes against lipid peroxidation and capable of reducing oxidized phospholipids in situ [55]. It was further verified that DHA stimulates a mechanism to self-protect from oxidative damage even under high aerobic conditions and elevated levels of transition metals, which inevitably favor the generation of reactive oxygen species, confirming our observations of a protective effect against rotenone-induced toxicity. Similarly, we demonstrated antioxidant proteins being upregulated in response to DHA pre-treatment, among them periredoxin-5, which is known to play a role in cell protection against oxidative stress [56] and is suspected to play an intricate role in the prevention of iron overload-induced neuronal death by mitochondrial fission [57]. The protective role of DHA could be further verified for mitochondria particularly since pre-treatment with DHA prior to the addition of Rot led to an overall increase in intensity for most mitochondrial proteins. Most notably, cells pre-treated with DHA displayed a significantly increased expression of proteins involved in the fatty acid beta oxidation (β-ox) and the citric acid cycle (CAC), suggesting a metabolization of the supplemented fatty acid by the cells.

**Figure 6:**
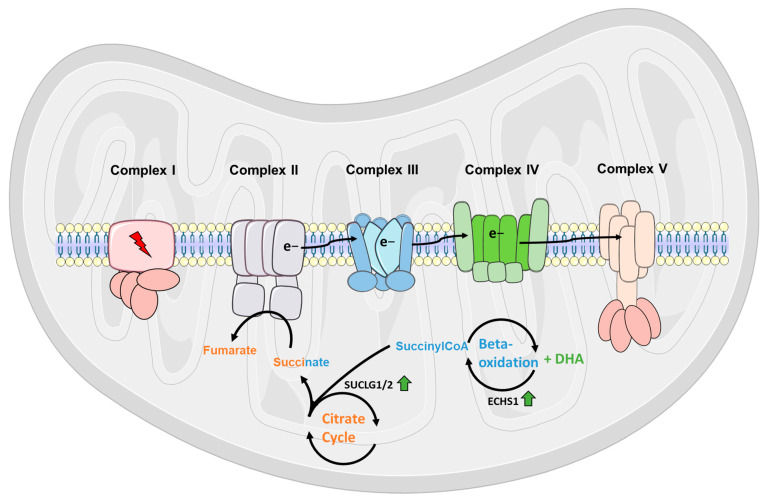
Proposed effect of DHA on rotenone stressed mitochondria. Supplementation of DHA results in its break down into succinyl-CoA via the fatty acid beta oxidation. In the citric acid cycle, succinyl-CoA is conserved into succinate by succinate-CoA ligases (SUCLG1/2) and subsequently converted into fumarate by succinate-dehydrogenase, which is both active in the citric acid cycle and the respiratory chain, functioning as complex II. (This figure was partially created using Servier Medical Art templates, which are licensed under a Creative Commons Attribution 3.0 Unported License; https://smart.servier.com, accessed on 29 November 2021).

Further, we hypothesize the following compensatory process: an increased activity of the β-ox and CAC may lead to an increased influx of electrons through complex II (see Figure 6), enabling a sufficient production of ATP, despite the inhibition of complex I. In detail, we identified the succinate-CoA ligases SUCLG1 and SUCLG2 to be uniquely upregulated in DHA pre-treated cells, which catalyze the conversion of succinyl-CoA to succinate, creating GTP from GDP. Succinyl-CoA is produced as an intermediate in the CAC but is also the end product resulting from the breakdown of long unsaturated fatty acids. Confirmatively, we identified the key protein of this step ECHS1, being of higher abundance in DHA pre-treated cells. Succinate may serve as an alternative electron donor, since it is converted into fumarate by succinate dehydrogenase, which is both active in the CAC and the respiratory chain, functioning as complex II. During the conversion, succinate is oxidised, while FAD oxidizes and carries the electrons to the first of the iron–sulfur clusters, creating an increased electron flux. This in turn may enable the active transport of protons via complex III and IV into the mitochondrial matrix and thus leading to a sufficient production of ATP in complex V. However, with our techniques, we cannot finally prove, whether pre-treatment with DHA truly changes the amount of ATP produced in comparison to Rot-stressed cells.

Overall, we can summarize, that DHA pre-treatment led to a protective effect in Rot-stressed cells and an increase in mitochondrial proteins, strengthening its promising role as safe and easy to administer supplement for therapeutic purposes. However, larger samples sizes and long-term treatment effects with DHA prior Rot administration should be considered and investigated to substantiate the presented results and to assess any potential adverse effects.

## Figures and Tables

**Table 1:** Unique significantly upregulated mitochondria-associated proteins identified via gene name in DHA + Rot-treated cells (n = 4) compared to Ctrl (n = 4). Significance is indicated by *p*-value and adjusted *p*-value (Student’s *t*-test, two-tailed, unpaired, adjustment according to Benjamini–Hochberg (q-value), differentially regulated proteins are defined by q-value < 0.05). Fold changes are depicted in DHA + Rot vs. Ctrl. Additional information on localisation and/or function is added.

Gene Names	*p*-Value	q-Value	Fold Change DHA + Rot vs. Ctrl	Localisation, Function
GLRX3	0.001	0.020	2.67	[2Fe-2S] cluster assembly, iron–sulfur cluster assembly
PDK1	0.001	0.022	2.01	regulation of glucose and fatty acid metabolism and homeostasis
GRPEL1	0.005	0.046	1.70	PAM complex, mitochondrial protein import
PRDX5	0.000	0.008	1.64	mitochondrial matrix, protection against oxidative stress
SUCLG2	0.003	0.032	1.62	citric acid cycle, hydrolysis of succinyl-CoA
ECHS1	0.002	0.022	1.53	mitochondrial fatty acid beta oxidation
ADCK3	0.003	0.035	1.43	biosynthesis of coenzyme Q
PHB2	0.001	0.012	1.42	mitochondrial integrity maintenance
HK1	0.002	0.025	1.40	mediates the initial step of glycolysis, cytosol and mitochondrial outer membrane
ACAT1	0.003	0.036	1.38	mitochondrial fatty acid beta oxidation
SUCLG1	0.004	0.044	1.30	citric acid cycle, hydrolysis of succinyl-CoA

## Data Availability

The mass spectrometry proteomics data have been deposited to the ProteomeXchange Consortium via the PRIDE partner repository with the dataset identifier PXD049302.

## Supplementary Materials

The following supporting information can be downloaded at: https://www.mdpi.com/article/10.3390/metabo15010029/s1, Supplementary tables: Table S1: Identified_proteins. Table S2: MitoCarta. Table S3: Differential_proteins. Table S4: Differential_proteins_overlap_GO_term. Supplementary figures: Figure S1: SILAC Labelling and Subcellular Fractionation. Figure S2: Quantitative Smart Protein Layers Western Blot analysis of mitochondrial marker protein cytochrome c oxidase (COX IV) in treated SH-SY5Y cells.
