# Supplementary material for: The Protective Effect of Docosahexaenoic Acid on Mitochondria in SH-SY5Y Model of Rotenone-Induced Toxicity
Source: Metabolites. 2025 Jan 8;15(1):29. doi: 10.3390/metabo15010029 (PMC11767228; doi:10.3390/metabo15010029)
Supplement: Supplementary file 1 [file metabolites-15-00029-s001.zip › Supplementary Figures_Description.pdf]

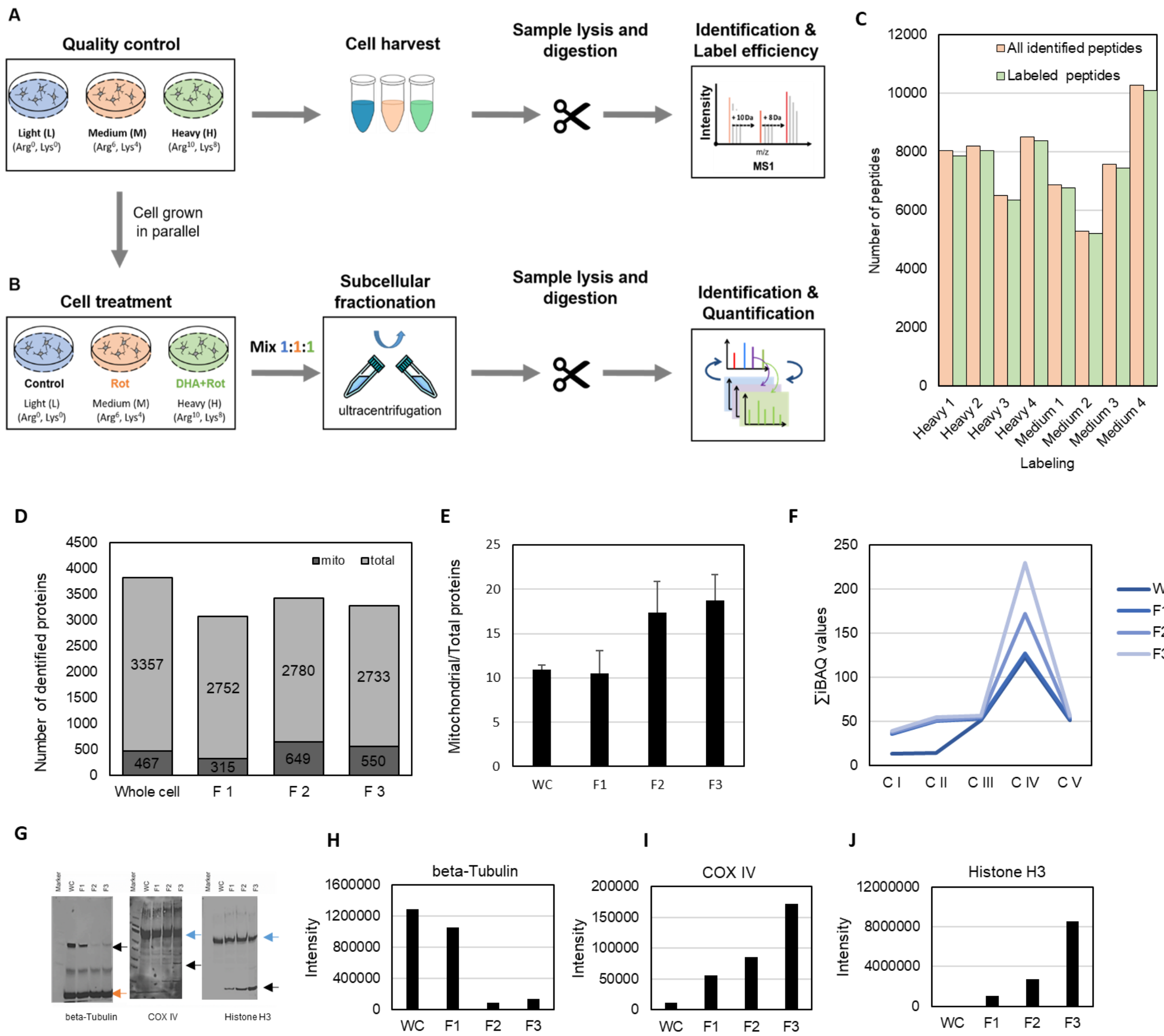

Supplementary Figure S1: SILAC Labelling and Subcellular Fractionation: A. Experimental design of the subcellular proteomic analysis of differentiated SH-SY5Y cells. Cells were stable isotopes labeled by amino acids in cell culture (SILAC) and lysed and digested (n=4). Afterward, the proteomes were measured and label efficiency was determined. **B.** Differentiated SH-SY5Y cells treated with Rot or a combination of DHA and Rot (n=5) Untreated cells were used as a reference control. The different groups were cultivated in different SILAC media. After cell collection, cells were mixed (1:1:1) and a simultaneous subcellular fractionation using density gradient ultracentrifugation was performed. The resulting fractions were collected, lysed, and tryptically digested. The peptides were analyzed using LC-MS/MS**. C**: Determination of labeling efficiency for medium and heavy labelled peptides. **D-F.** Comparison of proteins from subcellular fractionation to whole SH-SY5Y cells. **D.** Identified proteins and mitochondrial proteins represented as bar chart from whole cells and subcellular fractions **E.** Sum of normalized relative iBAQ intensities of mitochondrial proteins protein from whole cells and subcellular fractions. **F.** Profile plot of summed normalized relative iBAQ intensities of quantified proteins associated with Complex (C) I-V of the respiratory chain. **G)** SPL Western blotting system (NH DyeAGNOSTICS GmbH) was used for the detection of quantitative differences between selected candidate proteins. Total protein was pre-labeled with a red fluorescent fluorophore (700 nm), and a green fluorescence-labeled standard protein (orange arrow: S =12,5 kDa and blue arrow: size L = 80 kDa) was spiked in for error correction of differing sample loading and data normalization between experiments. Primary antibodies were used against beta-Tubulin, COX IV, and Histone H3 (Black arrow). **H-J)** SPL system analysis resulted in quantitative protein volumes (SPL normalized volume), for **H:** beta-tubulin **I:** COX IV and **J:** Histone H3.

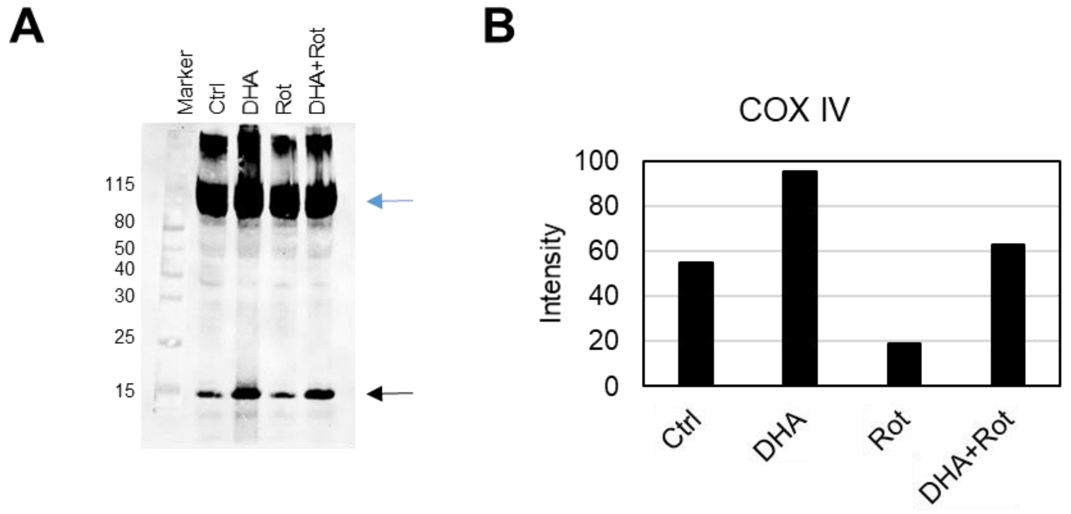

Supplementary Figure S2: “**Quantitative Smart Protein Layers Western Blot analysis of mitochondrial marker protein cytochrome c oxidase (COX IV) in treated SH-SY5Y cells. A**. SPL Western blotting system (NH DyeAGNOSTICS GmbH) was used for the detection of quantitative differences between COX IV in untreated SH-SY5Y cells (Ctrl), DHA and/or Rot-treated cells. Total protein was pre-labeled with a red fluorescent fluorophore (700 nm), and a green fluorescence-labeled standard protein Label L (~ 80 kDa indicated by blue arrow) was spiked in for error correction of differing sample loading and data normalization between experiments. Primary antibodies were used against COX IV (~ 12.5 kDa, indicated by black arrow). **B.** SPL system analysis resulted in quantitative protein volumes, which were normalized on the total protein loaded (SPL normalized volume). The abundance of COX IV was increased in DHA-treated cells, reduced in Rot-treated cells and DHA+Rot-treated cells similar to Ctrl.”
