# Supplementary figures and images for: The Protective Effect of Docosahexaenoic Acid on Mitochondria in SH-SY5Y Model of Rotenone-Induced Toxicity
Source: Metabolites. 2025 Jan 8;15(1):29. doi: 10.3390/metabo15010029 (PMC11767228; doi:10.3390/metabo15010029)

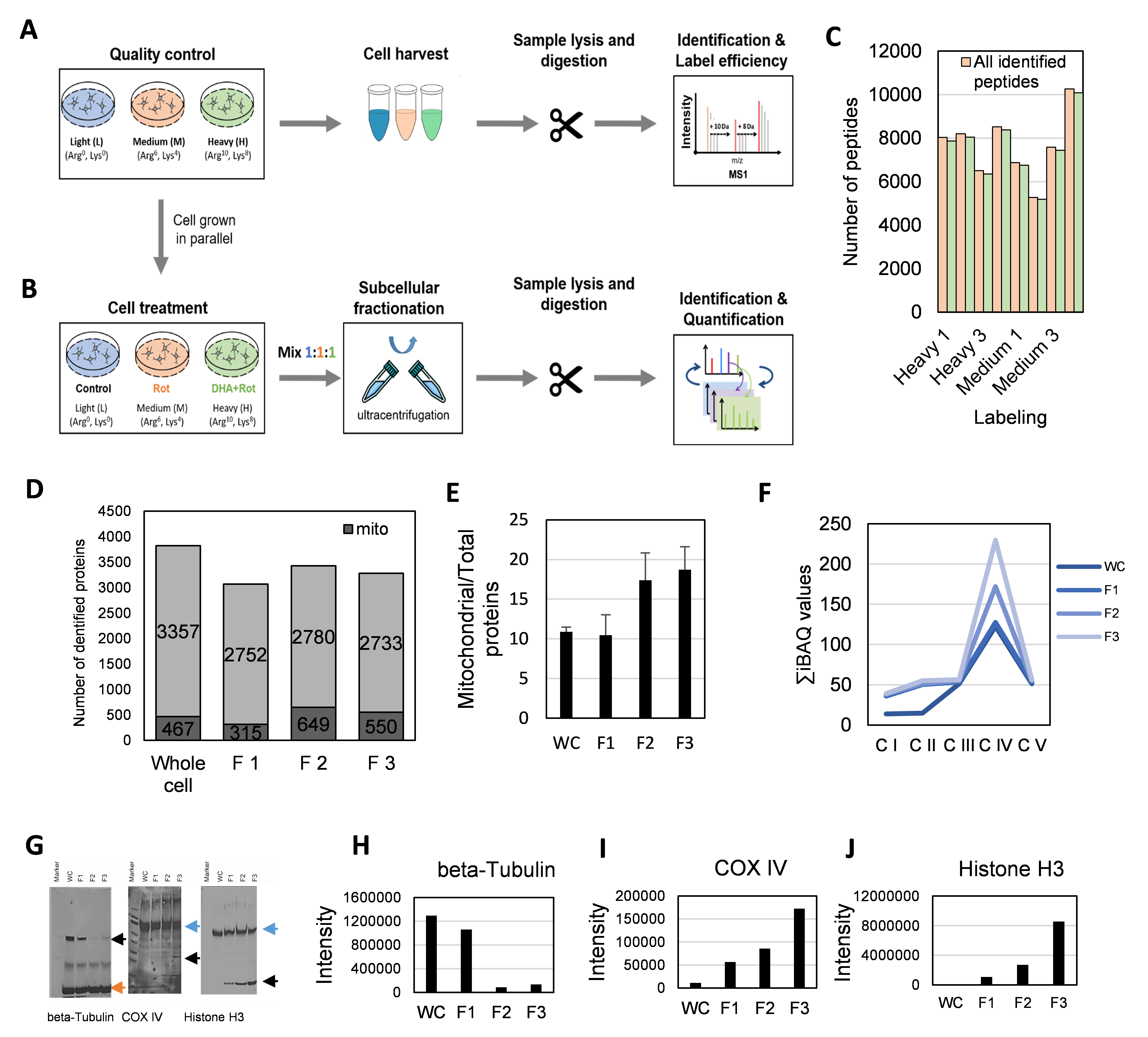

Supplement: Supplementary file 1 [file metabolites-15-00029-s001.zip › Supplementary_Figure_S1.tif]

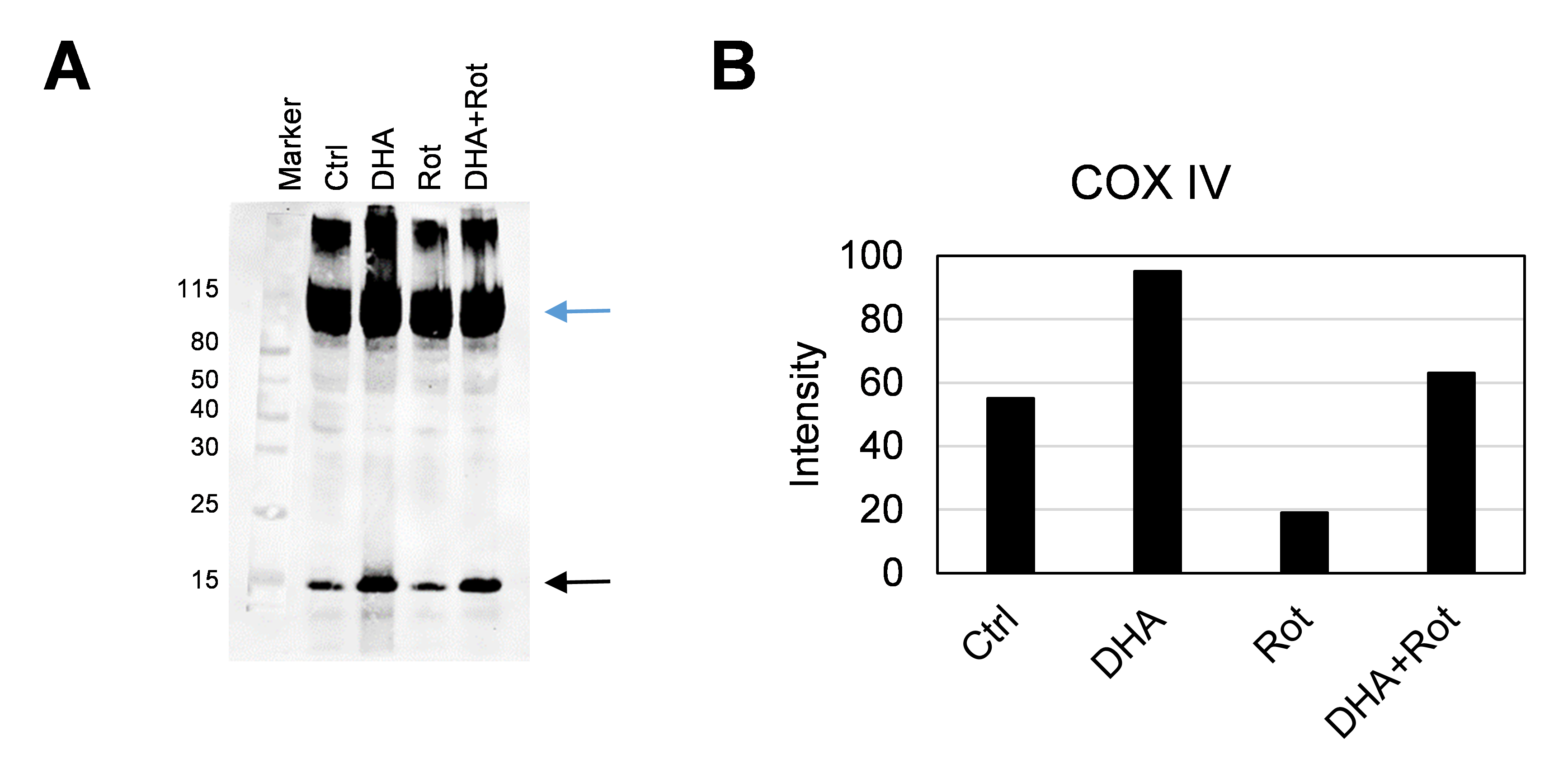

Supplement: Supplementary file 1 [file metabolites-15-00029-s001.zip › Supplementary_Figure_S2.tif]
